# Ellagic Acid Prevents α-Synuclein Spread and Mitigates Toxicity by Enhancing Autophagic Flux in an Animal Model of Parkinson’s Disease

**DOI:** 10.3390/nu16010085

**Published:** 2023-12-26

**Authors:** Nada Radwan, Engila Khan, Mustafa T. Ardah, Tohru Kitada, M. Emdadul Haque

**Affiliations:** 1 Department of Biochemistry and Molecular Biology, College of Medicine and Health Sciences, United Arab Emirates University, Al Ain P.O. Box 15551, United Arab Emirates; nadaa.radwan@outlook.com (N.R.); engila21@gmail.com (E.K.); mustafa_ardah@uaeu.ac.ae (M.T.A.); 2 Otawa-Kagaku, Parkinson Clinic and Research, Kamakura 247-0061, Japan; tohrukitada@gmail.com

**Keywords:** Parkinson’s disease, ellagic acid, PD mouse model, autophagy, α-synuclein

## Abstract

Parkinson’s disease (PD) is the second most common neurological disorder, pathologically characterized by loss of dopaminergic neurons in the substantia nigra pars compacta (SNc) as well as the formation of Lewy bodies composed mainly of α-synuclein (α-syn) aggregates. It has been documented that abnormal aggregation of α-syn is one of the major causes of developing PD. In the current study, administration of ellagic acid (EA), a polyphenolic compound (10 mg/kg bodyweight), significantly decreased α-syn spreading and preserved dopaminergic neurons in a male C57BL/6 mouse model of PD. Moreover, EA altered the autophagic flux, suggesting the involvement of a restorative mechanism meditated by EA treatment. Our data support that EA could play a major role in the clearing of toxic α-syn from spreading, in addition to the canonical antioxidative role, and thus preventing dopaminergic neuronal death.

## 1. Introduction

Polyphenols are among the most researched naturally occurring chemicals; their antioxidant capacity has attracted therapeutic potential in many pathologies [1,2]. One of the most prominent polyphenolic compounds is ellagic acid (EA), found in a plethora of dietary sources, including walnuts, cashews, cranberries, strawberries, raspberries [3,4] and pomegranates [5,6].

**Figure 1:**
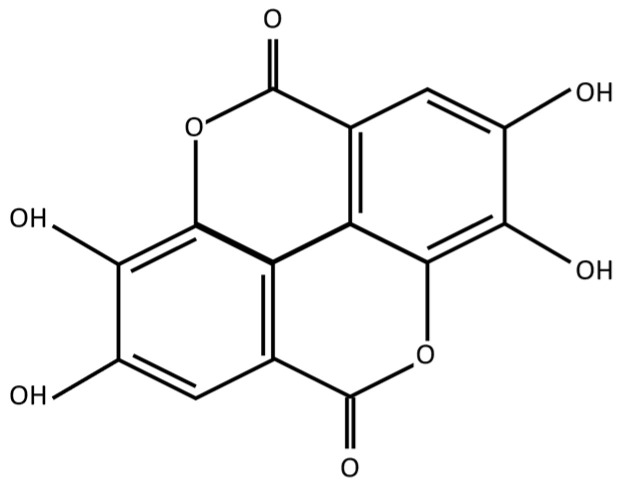
Ellagic acid molecular structure.

The phenolic groups present in EA (Figure 1) are hydrogen donors, conferring their antioxidant effect on reactive oxygen species as well as reactive nitrogen species, thus ameliorating harmful oxidative cellular stress. Furthermore, the presence of benzene rings creates a capacity to participate and buffer redox reactions [7].

The high antioxidative properties of EA has attracted a great deal of attention in various chronic diseases, many of which share inflammatory etiologies, such as cardiovascular disease [8], inflammatory bowel disease [9], respiratory syndromes [10] and liver diseases [11]. The antitumorigenic properties of EA have been extensively studied in many oncologic diseases, including pancreatic [12], lung [13], bladder [14], gastric [15], colorectal [16] and breast cancers [17], with the mitigation of adverse effects reported in multiple anticancer therapy studies [18,19,20].

Additionally, antiaging properties have been attributed to EA [21]; the significance of this property had been the primary focus in many neurological pathologies, especially in Parkinson’s disease (PD). PD is the second most common neurological disorder [22], of which the disease burden is the tenth-ranking neurological disorder in the 2019 Global Burden of Disease study [23]. PD clinically manifests in a series of motor symptoms, including tremors, rigidity, muscle stiffness and postural instability [24]. Although non-motor symptoms are documented to precede the clinically symptomatic onset of PD, the elusive nature of these non-motor symptoms pose a difficulty in the diagnosis of the disease [25]. The development of PD symptoms arises predominantly due to the lack of the neurotransmitter dopamine (DA). The depletion of DA occurs as a consequence of neuronal death of the dopaminergic neurons located in the midbrain, specifically in the substantia nigra pars compacta (SNc), which synthesizes it.

The neuropathology of PD had been highly associated with the accumulation of a presynaptic protein known as α-syn [26]. Although the physiological function of α-syn is proposed to influence the release of neurotransmitters, mapping of the α-syn function beyond pathological context is far from complete [27]. The aggregation of accumulated α-syn into toxic oligomers and fibrils [26,28], leading to the formation of insoluble inclusion known as Lewy bodies (LBs), is one of the hallmarks of PD [29]. The process of self-propagation of α-syn in a prion-like manner from afflicted to healthy neurons greatly explains Braak’s clinical staging of sporadic PD [30,31]. The templated α-syn aggregation is initiated in many PD models via the introduction of α-syn preformed fibrils (PFF) [32].

Many post-translational modifications (PTMs) of α-syn have been associated with PD pathology [33]; the presence of phosphorylated α-syn at Ser129 (pS129) in high ratios reaching up to 90% of LBs prompted the focus on pS129 α-syn detection in PD research [34].

The clearance of α-syn is primarily conducted via a catabolic process called autophagy (literally meaning ‘self-eating’). Induction of autophagic flux has been researched as a promising therapeutic strategy in many neurological pathologies [35]. In addition to the implication of EA in the betterment of PD prognosis, which has been primarily attributed to its antioxidative effect [36], the involvement of autophagy induction in EA action had been recently reported in a cell culture model of PD [37].

Earlier, we showed that EA effectively mitigates the acute neurodegenerative effects of 1-methyl-4-phenyl-1,2,3,6-tetrahydropyridine (MPTP) on dopaminergic neurons [38]. Initially identified in the early 1980s, MPTP’s neurotoxicity was first observed in young drug abusers exhibiting Parkinson-like symptoms [39]. MPTP’s ability to selectively target dopaminergic neurons in the SNc has significantly added to the capacity of animal modeling of PD [40]. MPTP, being lipophilic, easily crosses the blood–brain barrier (BBB). Inside the brain, it is metabolized by monoamine oxidase-B (MAO-B) into MPDP+, which then spontaneously oxidizes to form 1-methyl-4-phenylpyridinium (MPP+), a potent inhibitor of mitochondrial complex I. MPP+ disrupts the mitochondrial respiratory chain and calcium homeostasis, leading to increased reactive oxygen species (ROS) production and ATP depletion. The vulnerability of SNc dopaminergic neurons to MPP+ in various PD models is largely attributed to MPP+’s high affinity for dopamine uptake sites [41]. Elevated intracellular concentrations of MPP+ impair mitochondrial functions in these high-energy-demanding neurons, accelerating PD progression [42]. Our prior research revealed that IP administration of a nontoxic low MPTP dose (10 mg/kg b.wt.) exacerbated α-syn spreading and neurotoxicity in male C57BL/6 mice injected with PFF in the striatum [43]. The current study builds on that model, exploring how EA might prevent α-syn propagation and neurodegeneration.

## 2. Methodology

### 2.1. PFF Seed Synthesis

**Figure 2:**
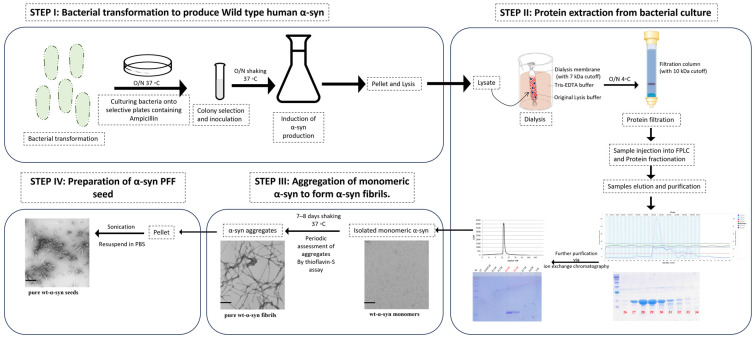
Human PFF seed synthesis.

PFF seed synthesis proceeded as described in [43], and is illustrated in Figure 2. Briefly, the first step proceeded by the bacterial transformation with pT7-7 wild-type α -syn vector and plating onto ampicillin selection plates to identify bacterial colonies successfully expressing the α-syn vector. The selection of one colony was inoculated in lysogeny broth (LB) overnight at 37 °C in the shaker. This colony was expanded into a larger LB volume and kept overnight, and optical density (OD) was regularly assessed until an OD of 0.5–0.6, at which exponential bacterial growth was achieved. Lac-repressor was inhibited by the addition of Isopropyl β-D-1-thiogalactopyranoside (IPGT) to the bacterial culture to induce wild-type human α-syn production (2 h at 37 °C in the shaker).

Bacterial culture was pelleted and lysed using a detergent-free lysis buffer. The lysate was subjected to dialysis using a gel filtration buffer (Tris-EDTA) and a dialysis tube with 7 kDa cutoff-molecular-weight buffer, which is compatible with the subsequent isolation techniques. Following dialysis, the lysate was subjected to filtration (using 2 µm filters), then was subjected to concentration (~10 kDa cutoff molecular weight). The concentrated lysate was subjected to fast protein liquid chromatography (FPLC). Fractionated protein samples were eluted, and molecular weight of the α-syn was collected with respect to the range of eluted fractions under the standard curve (peaks of chromatogram) detected. Further analysis of the range of fractions collected was analyzed using SDS-PAGE to ensure the collection of the best representative α-syn elution. The purity of the sample was further increased by subjecting collected fractions to ion exchange chromatography, through which α-syn monomer was isolated.

Monomeric α-syn was transformed into aggregated α-syn fibrils via subjecting to constant shaking (800 rpm/min) at 37 °C over a period of 7–8 days. Periodic testing of fibril formation was achieved using thioflavin-S assay. Furthermore, the visualization of the aggregatory phases of α-syn fibrils was confirmed using Western blotting and electron microscopy. Finally, the aggregated α-syn fibrils were pelleted and washed to remove trace monomeric α-syn. Application of the α-syn fibrils in pathological context requires a more interactive state, which is consistent with a smaller length of fibrils; this is achieved by the sonication of the aggregated α-syn fibrils to form α-syn PFF seed. This seed was then aliquoted and stored at −80 °C.

### 2.2. Animals

C57BL/6 mice were obtained from the UAE University animal facility. Male mice of ages 2–2.5 months that weighed 20–26 g were included in the experiments. Mice were housed in a 12 h light/dark cycle with access to food and water throughout the experiment duration. All experiments were performed in accordance with UAE University Animal Ethics guidelines (Approval number: ERA_2021_8408).

### 2.3. Study Plan

Male C57BL/6 mice were acclimatized to the polystyrene cages for a week; animal sedation was conducted using Ketamine HCL (Ketamil) and Xylazine HCL (Xylazil-20) solutions diluted in normal saline and dosed accordingly (1 mL/kg). Following sedation, the head was fixed onto the stereotaxic surgery apparatus (WPI) and a surgical scalpel blade (no. 11) was used to perform a cut (approximately from the sagittal suture to lambda), fully exposing the bregma. Following coordinates were used (with respect to the bregma) to deliver the PFF seed in the striatum (anterior–posterior: +0.5 mm; medial–lateral: −2.2 mm; dorsal–ventral: −3.4 mm) [43]. Intrastriatal injection (using Hamilton 10 μL syringe) was conducted at the following infusion rate: 0.5 μL/min using a syringe pump (Micro4 ^TM^ MicroSyringe Pump Controller, WPI, Sarasota, FL, USA); PFF infusion of 2.5 μL (a total of 5 μg of PFF seed) was delivered at a constant rate (0.5 μL/min) and an extra one minute was kept to allow for the remnant PFF seed in the syringe to diffuse properly. Following the stereotaxic surgery, wound was sealed by application of a tissue adhesive (3M Vetbond^TM/MC^, Kowloon, Hong Kong) onto the wound edges that had been clamped together using a forceps. Phosphate-buffered saline (PBS) group was kept as a control.

Intraperitoneal (IP) administration of the natural compound of interest (Ellagic acid-E2250-Sigma, St. Louis, MO, USA) proceeded for the following 12 days. EA was prepared in a 20 mg/mL stock in Dimethyl sulfoxide solvent (DMSO), then aliquoted into 200 μL aliquots and stored in −80 °C. Each aliquot of EA was mixed in saline solution in a 1:20 ratio to achieve final dose of 10 mg/kg b.wt. A 4-week gap was given before proceeding with the neurotoxin (MPTP-M08896-Sigma, St. Louis, MO, USA) IP administration at 10 mg/kg b.wt. for 5 consecutive days. The experimental group received the EA at least one hour before MPTP and continued for seven days. Control groups receiving EA vehicle (V): PFF+MPTP+V and PBS+V were given an IP 1:20 ratio of DMSO in saline solution for the same duration as EA treatment.

**Figure 3:**
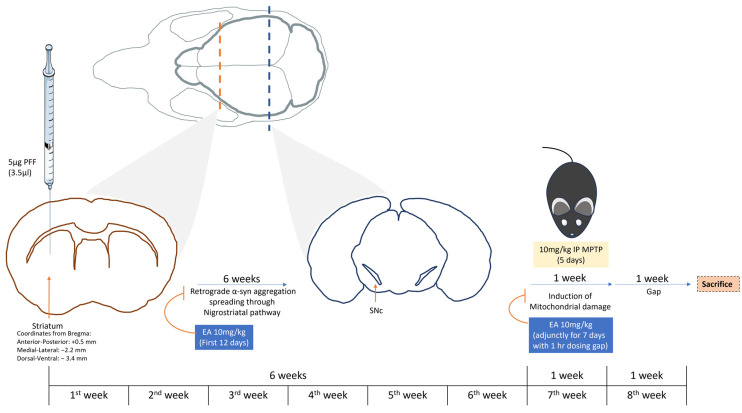
Study plan. Illustration of the study depicting the study plan and duration. C57BL/6 male mice were subjected to intrastriatal injection with PFF at coordinates: anterior–posterior: +0.5 mm; medial–lateral: −2.2 mm; dorsal–ventral: −3.4 mm from the bregma. Following 6 weeks, MPTP was administered IP at a low dose (10 mg/kg b.wt.) for 5 days. Animal sacrifice and brain collection were performed on the end of the 8th week of the experiment. IP EA administration (10 mg/kg b.wt.) was conducted during the first 12 days following the stereotaxic surgery and during the 7th week (adjunctively with MPTP administration with 1 h dosing gap) for 7 days.

Animal sacrifice was performed at the end of the 8th week of the experiment, as illustrated in Figure 3. Animals were injected with sedative, as mentioned before. The cardiac perfusion was conducted using 10 mL of normal saline followed by 10 mL of 4% Paraformaldehyde solution for tissue fixation. The collected brains were kept in 4% Paraformaldehyde solution for 24 h; for the following 3 days, this solution was changed twice a day with a 10% sucrose in 0.1 M PB + 0.02% sodium azide solution. Finally, the brains were carefully dried and frozen at −80 °C. Cryosectioning of the brain samples was performed at −20 °C. Brains were cut into 40 μm sections. Sections of striatal and midbrain (SNc) regions were saved in a serial manner in PBS 0.02% sodium azide (free floating), respectively.

### 2.4. Sample Processing

The assessment of the pathological features of the PD model with and without EA treatment in this study was evaluated via immunohistochemistry (IHC), immunofluorescence (IF) and sodium dodecyl-sulfate polyacrylamide gel electrophoresis (SDS-PAGE) assays. The following sections will further illustrate the methodologies implemented. Table 1 highlights the antibodies used in the IHC and IF assays in this study with their respective dilutions. Section collection for further tissue processing was performed as previously described in [43,44].

#### 2.4.1. DAB (3,3′-Diaminobenzidine) Stain

DAB stain was performed to the collected striatal sections to assess the dopaminergic nerve terminal (DAT) loss; collection of 7 the striatal sections (from the serially collected section area from −1.54 to −0.22 mm of bregma) of the free-floating 40 µm sections of the brain samples was conducted. These samples were washed in PBS, pH 7.4 in 5 min incubations 3 times. Following these washes, sections were blocked to minimize background signal with 10% normal goat serum (NGS) in PBS for 1 h at room temperature. PBS washes (5 min incubations 3 times) was performed followed by incubation with the primary antibodies overnight at −4 °C. The sections were kept at room temperature for at least 20 min before further processing to avoid tissue damage, primary antibody was washed with standard PBS washes (5 min incubations 3 times) then incubated with secondary antibody (Biotin conjugated) for 1 h at room temperature to increase the signal specificity as well as intensity. PBS washes were performed prior to incubation with tertiary antibody (Streptavidin-HRP Conjugate) for 1 h at room temperature, followed again by PBS washes. Signal acquisition was performed by DAB reaction.

Sections were mounted on the slides and dehydrated by exposure to serial dilutions of ethanol. Shandon synthetic mounting media (Ref 6769007) was then used for sealing. Image acquisition was performed using Leica DM4000 B LED Microscope (Leica Microsystems, Wetzlar, Germany). Optical density of DAT was measured using NIH Image J (version: Image J 1.54d).

DAB stain was also performed to the collected SNc sections to assess neuronal cell body loss using tyrosine hydroxylase (TH) positive neurons counting.

SNc sections serially collected from areas −2.18 to −3.80 mm with respect to the bregma were considered for this assay; a total of 7 sections (with a consistent 6-section interval) were collected for further tissue processing. The protocol proceeded in the same manner as DAT stain with the compatible secondary and tertiary antibodies to TH antibody, as detailed in Table 1. The TH counting proceeded as previously described in [38], using optical fractionator of automated stereo investigator (version 2018).

#### 2.4.2. Immunofluorescence (IF)

Immunofluorescent double labeling of the collected brain samples’ sections was performed to assess the following aspects in our study:

- α-syn species spreading to the dopaminergic neurons in the SNc in phosphorylated form (pSer129) and filamentous form (Proteinase K resistant form).
- Expression of autophagic markers (LC3, p62) in dopaminergic neurons in the SNc.

Sample collection of SNc proceeded in the same manner as the TH-DAB stain protocol to detect the following co-stains: TH-pS129, TH-Confirmational α-syn, TH-LC3 and TH-p62, respectively. The collected sections were blocked and washed as detailed in the DAT staining. Primary antibodies were diluted in PBS following the dilutions mentioned in Table 1 overnight at −4 °C. The sections were kept at room temperature for at least 20 min before further processing to avoid tissue damage; primary antibody was washed with standard PBS washes then incubated with florescent secondary antibody (TH was tagged with correspondent alexa594, while pS129, confirmational α-syn, LC3 and p62 were tagged with correspondent alexa488, respectively) for 1 h at room temperature and covered with aluminum foil to avoid photobleaching. Following 3 PBS washes (5 min each), sections were mounted onto the slides and sealed using Fluoroshield (F6182, Sigma, St. Louis, MO, USA).

TH-conformational α-syn co-stain required exposure to Proteinase K (PK) 5 µg/mL for 30 min at 25 °C prior to the blockade with 10%NGS and proceeded as other stains.

#### 2.4.3. TUNEL Assay (Terminal Deoxynucleotidyl Transferase dUTP Nick End Labeling)

In situ cell death detection kit (Roche Cat. No. 11 684 817 910) was used in this assay, Proteinase K (PK) 5 µg/mL for 30 min at 25 °C, prior to the blockade, and proceeded as per manufacturer recommendations. Nucleus was stained using TO-PRO^TM^-3 Iodide (642/661, Invitrogen); TOPRO (originally blue) was pseudo-colored to magenta.

#### 2.4.4. Western Blot Analysis

To confirm the data acquired in the IHC, sectioned samples of SNc were collected in a serial manner similar to the IHC and IF protocols in 0.1% Triton solution with 1× Protease Inhibitor cocktail in a 0.5 mL Eppendorf tubes, respectively. Tubes were sealed with parafilm and placed on a rotator (25 rpm) overnight at 4 °C.

Samples were completely homogenized by sonicating on ice (Sonic Ruptor 250-Omni international Homogenizer Company, Kennesaw, GA, USA) before centrifugation at 14,000 rpm 4 °C for 15 min. Samples were processed in 15% SDS-Poly acrylamide gel. Protein transfer was performed using wet transfer method onto methanol-activated PVDF membrane at 100 V for 1.5 h. Membrane was later blocked using 5% milk in PBS-T (or 5% bovine serum albumin in case of detection of phosphorus-containing protein). Membranes were tagged with the primary antibody (in dilutions detailed in Table 2) overnight at 4 °C on the shaker. PBS-T washes for 30 min preceded 1 h incubation with the correspondent secondary antibody. Bands were visualized using Sapphire (1.3.0219.0) azure biosystems.

### 2.5. Microscopy

Confocal microscope Nikon EZC1 was used in the image acquisition of colocalizing fluorescent markers (TH-pS129, TH-conformational α-syn, TH-LC3, TH-p62 and To-Pro-TUNEL, respectively). Leica DFC 3000 G microscope was used in the acquisition of the DAB striatal DAT signal.

Stereology of dopaminergic neuronal bodies (TH expressing cells in the SNc are visualized by DAB reaction) was performed using an optical fractionator of automated stereo investigator, as previously described in [38].

### 2.6. Data Acquisition and Statistical Analysis

NIH ImageJ software was used in the analysis of IF colocalization studies as well as DAB signal intensity measurement and the Western blotting band intensity study. GraphPad prism 5.0 software was used for statistical analysis. The number of animals is indicated by (n), where n = 3–4 animals per group for all experimental procedures.

## 3. Results

### 3.1. EA Inhibits MPTP-PFF Induced α-Syn Spreading and Aggregation

In vitro data suggest the candidacy of EA in the prevention of α-syn aggregation [45]. The propagation of α-syn aggregate spreading from the site of the primary PFF inoculation (striatum), where dopaminergic neurons terminals reside, to the cell body in the SNc, occurs in a retrograde manner across the nigrostriatal pathway [32].

**Figure 4:**
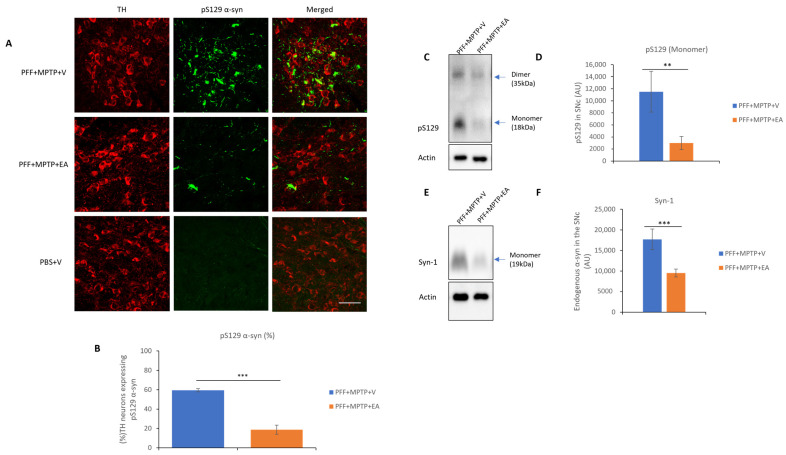
Assessment of α-syn in TH neurons in SNc. (**A**) Representative images showing the mouse pS129 α-syn spreading in the SNc in both PFF+MPTP-treated groups in comparison with a control group (PBS). (**B**) EA administration confers significant reduction in synuclein-spreading C57BL/6 male mice PD model. (**C**) Western blot showing phosphorylated form of α-syn in the SNc. (**D**) Western blot analysis showing phosphorylated form of α-syn a primary component found in Lewy bodies, and results were quantified as density relative to actin protein expression. (**E**) Western blot of endogenous levels of α-syn in SNc. (**F**) Quantification of endogenous α-syn protein. Data represented as percentage ± standard error of the mean (n = 3–4 per group). Scale bar: 50 µm (**A**). (*** *p* < 0.0001, ** *p* < 0.001, one-way ANOVA and Bonferroni post hoc test) (AU: arbitrary unit).

**Figure 5:**
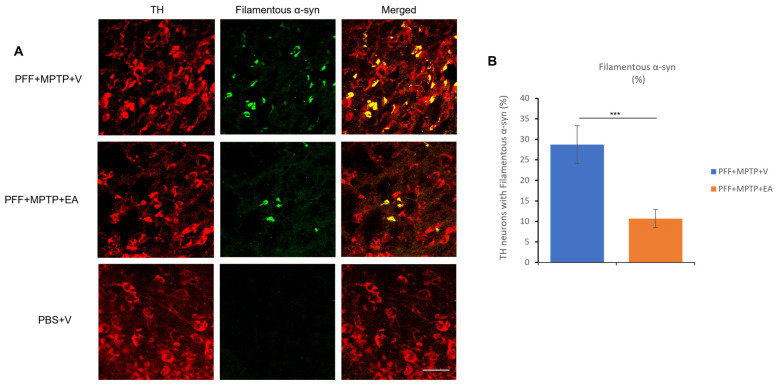
Assessment of filament-specific α-syn in the TH neurons of SNc. (**A**) Representative images showing the accumulation of filament-specific α-syn in the SNc in both PFF+MPTP-treated groups (PFF+MPTP+V and PFF+MPTP+EA), respectively. (**B**) EA administration confers a significant reduction in aggregated synuclein accumulation in C57BL/6 male mice. Data represented as percentage ± standard error of the mean (n = 3–4 per group). Scale bar: 50 µm (**A**). (*** *p* < 0.0001, one-way ANOVA and Bonferroni post hoc test).

This study, through the recently developed in vivo model in male C57BL/6 mice [43], follows the propagation of α-syn in the presence and absence on EA treatment (IP 10 mg/kg b.wt.). The spreading and aggregation of α-syn was determined via the evaluation of two subsequent antibody stains. The first was through the detection of the pS219 α-syn (Figure 4A), which is the most common form of PTM of α-syn found in LB pathology in PD patients. And the second test was the detection of a PK-resistant form of α-syn, commonly referred to as conformational or filamentous α-syn, which is more consistent with pathogenic fibrillar α-syn structures (Figure 5A).

EA produced a significant reduction in the colocalization of both pS219 α-syn and filamentous α-syn in the TH neurons, as represented in Figure 4B and Figure 5B, respectively. Collectively, these results suggest the inhibitory effect of EA on α-syn spreading, as well as removing the α-syn aggregates in vivo.

### 3.2. EA Inhibits Dopaminergic Neuronal Cell Loss and Preserves DAT Terminals

EA conferred neuronal protection against α-syn spreading and aggregation in C57BL/6 male mice, a neuroprotective feature that is in concordance with EA in vitro action [37,38,46]. The preservation of the dopaminergic neurons was achieved by the administration of IP 10 mg/kg dose of EA over the 12 days that followed the stereotaxic surgery in addition to an adjunctive administration with the neurotoxin MPTP (with a 1 h dosing gap), as mentioned in the methods and materials section.

**Figure 6:**
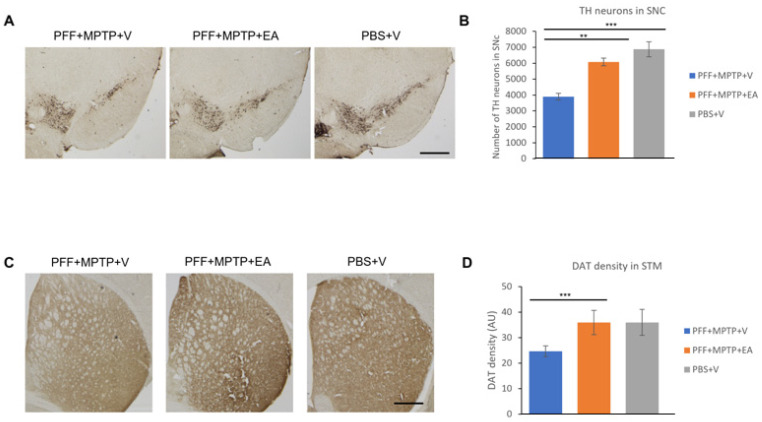
Assessment of tyrosine hydroxylase immune-positive (TH+) neurons to quantify the number of dopaminergic (DA) neurons in the substantia nigra (SNc) and dopamine transporter (DAT) density in the striatum. (**A**) Representative images showing the TH+ neurons in the SNc area. (**B**) The number of DA neurons in the SNc was counted in each animal using unbiased stereo investigator system, as described in methods section. The number of TH+ neurons was significantly higher in animals treated with EA. (**C**) Representative images showing the immunoreactivity of dopamine transporter (DAT) in the striatum. The intensity of dopamine nerve terminals was significantly improved in the striatum of EA-treated male mice when compared with that in group (PFF+MPTP+V). (**D**) DAT intensity was measured using NIH image J software, and is presented in the graph. Scale bar: 500 µm (**A**,**C**). Data represented as percentage ± standard error of the mean (n = 3–4 per group). (*** *p* < 0.0001, ** *p* < 0.001, one-way ANOVA and Bonferroni post hoc test) (AU: arbitrary unit).

An unbiased stereoinvestigator system was used to assess the TH neuron number in the SNc area. Through stereoscopy, significant preservation of dopaminergic neurons in EA-treated animals when compared to the vehicle-treated group was observed, as displayed in Figure 6B, which highlights of the neuroprotective role of EA.

Since DA neurons project their nerve terminal to the striatum area, any loss of DA neurons results in a decrease in the number of DATs. The assessment of this preservation was achieved using DAB staining and by measuring the intensity of the nerve terminals. As expected, significant preservation of DAT intensity in the EA-treated group as opposed to vehicle group was observed (Figure 6D).

### 3.3. EA Enhances the Apoptotic Profile of Neurons in the SNc in MPTP-PFF Mouse PD Model

**Figure 7:**
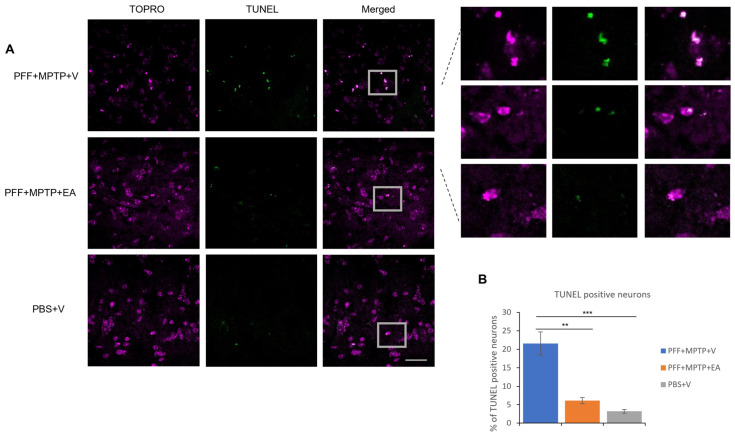
Detection and quantification of apoptotic neurons using TUNEL assay. (**A**) Representative images showing the TUNEL-positive neurons in the midbrain sections covering SNc region. EA treatment confers significant protection against apoptosis when compared with (PFF+MPTP+V) group; nuclei stained with TOPRO (originally blue) were pseudo-colored to magenta. (**B**) Number of TUNEL-positive cells counted and presented. Data represented as percentage ± standard error of the mean (n = 3–4). Scale bar: 25 µm. (*** *p* < 0.0001, ** *p* < 0.001, one-way ANOVA and Bonferroni post hoc test).

As a hallmark of apoptosis, TUNEL labeling was performed on the SNc neurons. As displayed in Figure 7B, the EA-treated group displayed a significantly lower presence of nuclear fragmentation, which is consistent with the previous results ensuring the neuroprotective effect of EA treatment against the neurotoxicity of α-syn aggregate spreading from the striatum to the SNc, as well as the MPTP neurotoxin.

### 3.4. EA Induces Autophagic Degradation of α-Syn

**Figure 8:**
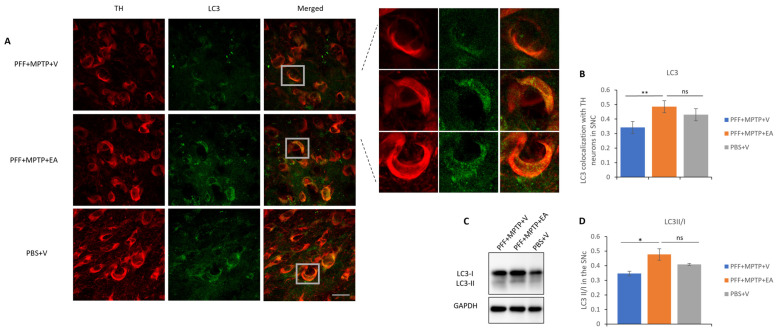
Assessment of autophagy marker LC3 in TH neurons in the SNc area. (**A**) Representative images showing the LC3 puncta in the SNc area. (**B**) EA significantly increases LC3 puncta colocalized in the TH neurons of the SNc area as compared with vehicle-only-treated group (PFF+MPTP+V). Data represented as Pearson’s coefficient ± standard error of the mean (n = 3–4) as mentioned in the methods and methodology. Scale bar: 25 µm (**A**). (**C**) Immunoblotting of autophagy marker LC3 in both its isoforms, LC3II and LC3I. (**D**) An increase revealed in the LC3II/I ratio in the EA-treated group as opposed to the group without EA treatment. Data represented as LC3 II/I ratio ± standard error of the mean (n = 3–4). (** *p* < 0.001, * *p* < 0.05, ns: not significant, one-way ANOVA and Bonferroni post hoc test).

The autophagic lysosomal pathway is one of the main mechanisms responsible for the degradation and clearance of α-syn; therefore, a colocalization study between the TH neurons and the LC3 protein was performed. It was found that EA-treated samples exhibited significantly higher levels of colocalization of the LC3 protein in the TH neurons consistent with displayed inhibition of α-syn spreading and aggregation as well as preserved structural integrity of dopaminergic neurons. Figure 8B displays the increased LC3 marker in TH neurons in the SNc upon EA treatment. These data were further confirmed by the assessment of the autophagy flux via the quantification of the LC3 II/I ratio by SDS-PAGE assay, as illustrated in Figure 8C,D.

**Figure 9:**
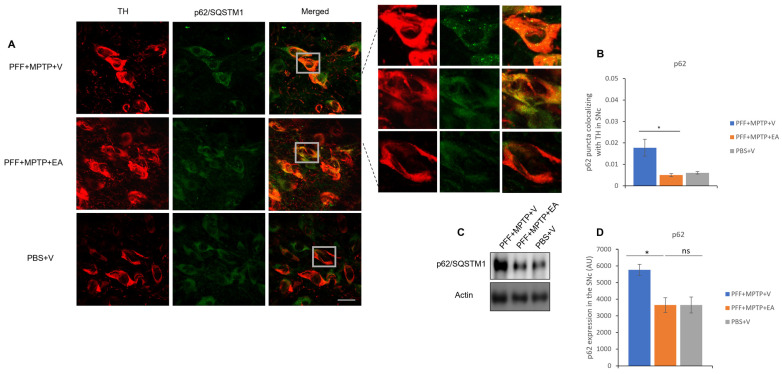
Assessment of autophagy substrate p62 in TH neurons in the SNc. (**A**) Representative images showing the p62 puncta in the SNc. (**B**) EA significantly increases p62 puncta colocalized in the TH neurons of the SNc as compared with vehicle-only-treated group PFF+MPTP+V). (**C**) Western blot showing significant decrease in p62 level when animals were treated with EA. (**D**) Quantification and analysis of immunoblot signal using NIH image J software. Data represented as Pearson’s coefficient ± standard error of the mean (n = 3–4). Scale bar: 25 µm (**A**). (* *p* < 0.05, ns: not significant, one-way ANOVA and Bonferroni post hoc test) (AU: arbitrary unit).

It is noteworthy that assessment of the molecular chaperone, p62, is essential in the recruitment of autophagic biomarkers and cellular cargo, thus aiding in the facilitation of the catabolic process through lysosome. The p62 is a substrate of the autophagic flux; in other words, its accumulation is a signature of dysfunctional autophagy. Colocalization showed to be decreased, with a smaller number of p62 inclusions that colocalized with TH neurons in the SNc of the EA-treated groups in comparison with the control group (as displayed in Figure 9B). Western blot analysis verified the capacity of EA to restore normal autophagy in a PD mouse model (Figure 9D).

## 4. Discussion

PD is the second most common neurodegenerative disorder, marked by the depletion of dopaminergic neurons in the motor control center of the midbrain (specifically in the SNc). The deposition of aggregated presynaptic protein α-syn is one of the hallmarks of the disease, and is seen in neurons [30]. The development of α-syn aggregates from a nontoxic monomer via toxic oligomeric states into a relatively less toxic fibrillar state as the primary component of LBs is the central dogma of loss of synaptic plasticity in PD. pS129 α-syn composes up to 90% of the LBs α-syn content [33,34]. The association of the pS129 in the LB development polarized detection of this form of α-syn for the progression of PD neuropathology in many experimental models [47].

The clearance of α-syn is predominantly carried out via a catabolic cascade known as autophagy. The most investigated participants of this cascade are LC3 (Microtubule-associated protein 1A/1B-light chain 3) and p62 (p62/Sequestosome 1). LC3, on one hand, presents the gold standard in the autophagy flux detection, as it exists as an LC3 I isoform, which is lipidated to form LC3 II upon active autolysosomal degradation of cellular cargo [48]. p62, on the other hand, is a substrate of autophagy, which chaperones autophagy biomarkers and ubiquitinates cellular cargo for autolysosomal degradation [49].

The current therapeutic approach to PD is primarily dopamine replacement therapy, not curative therapy. The approaches to expand the scope of PD therapies to target α-syn accumulation as well as the neuroinflammatory component of the disease have been the primary target in PD research. Many natural products with antioxidative capacity are being utilized in the approach to develop PD therapy that targets neuronal loss rather than the conventional supplementation of neurotransmitter deficiency [50].

EA, a naturally occurring polyphenol, is among the most studied antioxidants that have gained wide recognition in the field of nutraceuticals [51]. Owing to its antioxidant capacity, EA had been proposed as a nutraceutical in many chronic diseases [9,10,52].

Although EA, like many polyphenolic compounds, displays limited oral bioavailability [53] and extensive phase II metabolism [54], consumption of EA showed efficiency in clinical trials involving multiple maladies [52,55,56], some of which are of a neurological nature [57,58]. The safe pharmacokinetic profile of EA [59,60] favors research aiming to enhance EA bioavailability via improved drug delivery systems [61].

In vitro data verified the direct physical intervention of EA in the aggregation process of α-syn. An in vitro cellular model of PD confirmed a negative correlation between α-syn aggregation and EA treatment [37]. Furthermore, the polyphenolic compound was found to stimulate anti-inflammatory pathways in multiple animal models of PD, thus confirming the therapeutic potential of EA. The aim of this study was to test the efficacy of EA in our recently developed in vivo model of PD [43]. Evaluation of the EA role limiting the α-syn spreading across the nigrostriatal pathway and neurotoxicity in vivo was conducted. Additionally, this study investigates the potential role of EA-induced autophagic stimulation, which could play a role in α-syn aggregate degradation.

Enhancement of α-syn aggregate formation was previously reported upon PFF seed injection in the striatum when a low dose of MPTP is administered [43]. Therefore, retrograde spreading of α-syn aggregates from the site of administration (striatum) to the dopaminergic neuron soma (SNc) was assessed by the detection of pS129 α-syn (Figure 4C), endogenous α-syn (Figure 4E) and conformational α-syn antibodies (Figure 5A). EA treatment significantly decreased α-syn expression in the SNc (as displayed in Figure 4D,F and Figure 5B), thus extending the validity of in vitro and cell culture data to the animal model system.

Further exploration of the integrity dopaminergic neurons yielded positive results; EA conferred protection against dopaminergic neuron loss (Figure 6A), detected by the staining of the neuronal cell body in the SNc (using TH, a rate limiting enzyme in the dopamine synthesis) as well as the nerve terminals (Figure 6C) in the striatum (using DAT, a critical biomarker for synaptic integrity and plasticity). These results were further substantiated by the assessment of the apoptotic neurons in the SNc via the detection of neuronal DNA fragmentation (primarily associated with apoptosis) using TUNEL assay. EA significantly prevented neuronal cell death in the SNc (Figure 7A), which is concordant with previous data confirming protection against dopaminergic neuron loss in the SNc tested by DAB assay TH staining.

Investigation of the effect of EA treatment on autophagic markers was performed in this study. EA increased autophagy marker LC3, as confirmed by the increased colocalization of LC3 protein with the TH-expressing neurons in the SNc (Figure 8A) as well as the increased LC3 II/I ratio (Figure 8D). Furthermore, a significant decrement in the accumulation of p62 in both immunofluorescence (Figure 9A) as well as Western blot analysis (Figure 9C) was observed. Increased neuroprotection upon EA IP administration in the PD mouse model in this study could be attributed to the enhancement of the autophagic flux, which plays an important role in the lysosomal degradation of α-syn as well as the targeting of dysfunctional mitochondria [62,63]. Collectively, these results suggest the effect of EA IP administration in the male mouse model of PD in restoring functional autophagic flux in SNc dopaminergic neurons, thus providing neuroprotection.

A very recent article has showed that knockout of UPS30 or the selective inhibitor of UPS30 is beneficial as a disease-modifying target that enhances the clearance of defective mitochondria induced by aggregated synuclein [64]. It has been suggested that the suppression of USP30 may have a significant impact on mitochondrial morphology, maintenance and function, and thus, risk associated with its suppression should be noted carefully. The toxicity of α-syn causes damage not only in the mitochondria, but also in the nucleus and the cytoplasm as well as at the synaptic level [65,66]. Furthermore, the properties of EA are well known, and its safety is well established [59]. In that context, EA is more promising because of its role as an antioxidant, inhibiting synuclein aggregation and promoting autophagy.

In this study, we specifically included only male mice, aligning with epidemiological data that indicated a higher PD incidence among males [67]. However, considering that PD progression rates are reportedly higher in females [68], it is crucial to extend our investigation to female mice in future research. This will help in understanding any gender-specific responses to EA treatment. Additionally, future studies should be designed to explore into the molecular mechanisms underlying the induction of autophagy by EA. Understanding these mechanisms is key to developing effective treatments. Lastly, from a translational research standpoint, exploring oral administration of EA and its potential benefits in PD models warrants further attention. Such studies could pave the way for more accessible and noninvasive treatment options for PD patients.

## 5. Conclusions

In the current study, we were able to prove the neuroprotective capacity of EA in improved PD mouse model. EA treatment significantly reduced α-syn spreading and neurotoxicity. Induction of the autophagic flux, as well as suppression of apoptosis, in SNc neurons was confirmed in this study, shedding light on a potential therapeutic mechanism of action mediated by EA in a PD mouse model.

## Figures and Tables

**Table 1:** IHC antibodies.

Antibodies	Host Species/Cat. No.	Source	Dilution	Assay
**Primary Antibodies**				
DAT	Rat/TEMECULA MAB369	Merck, Burlington, MA, USA	1:1000	DAB
TH	Mouse/Immuno star 22941	ImmunoStar, Hudson, Wisonsin, USA	1:1000	DAB/IF
pS129 α-syn	Rabbit/ab59264	Abcam, Waltham, MA, USA	1:1000	IF
Conformational α-syn	Rabbit/ab209538	Abcam, Waltham, MA, USA	1:1000	IF
LC3A/B	Rabbit/CST 12741	Cell Signaling Technology, Inc., Danvers, MA. USA	1:500	IF
p62	Mouse/ab56416	Abcam, Waltham, MA, USA	1:500	IF
**Secondary Antibodies**				
Biotin-sp-conjugated	Donkey anti-Rat/Jackson Immuno Research 712-065-153	Jackson ImmunoResearch Laboratories, West Grove, PA, USA	1:1000	DAB
Biotin-sp-conjugated	Donkey anti-mouse/Jackson Immuno Research 715-065-150	Jackson ImmunoResearch Laboratories, West Grove, PA, USA	1:1000	DAB
Alexa Fluor 594	Goat anti-mouse/Invitrogen; A11032	Thermo Fisher Scientific Pierce Biotechnology, Rockford, IL, USA	1:1000	IF
Alexa Fluor 488	Goat anti-rabbit/Invitrogen; A11034	Thermo Fisher Scientific Pierce Biotechnology, Rockford, IL, USA	1:1000	IF
**Tertiary Antibody**				
Streptavidin-horseradish Peroxidase conjugate	AntiDonkey/Amersham^TM^; RPN1231-2ML	Cytiva, Buckinghamshire, UK	1:200	DAB

**Table 2:** Western blot antibodies.

Antibodies	Host Species/Cat. No.	Dilution
TH	Mouse/Immuno star 22941	1:1000
pS129 α-syn	Rabbit/ab59264	1:1000
LC3A/B	Rabbit/CST 12741	1:1000
p62	Mouse/ab56416	1:1000
GAPDH	Rabbit/CST 2118	1:1000
Actin	Mouse/MAB1501R	1:1000
Goat Anti-rabbit	Goat/Jacson Immuno Research 111-035-144	1:10,000
Goat Anti-mouse	Goat/Jacson Immuno Research 115-035-166	1:10,000

## Data Availability

The data presented in this study are available on request from the corresponding author. The data are not publicly available due to privacy.
